# Supplementary material for: Pan-genomic matching statistics for targeted nanopore sequencing
Source: iScience. 2021 Jun 8;24(6):102696. doi: 10.1016/j.isci.2021.102696 (PMC8237286; doi:10.1016/j.isci.2021.102696)
Supplement: Document S1. Figures S1–S5 and Table S1 [file mmc1.pdf]

iScience, Volume *24*

Supplemental information

Pan-genomic matching statistics

for targeted nanopore sequencing

Omar Ahmed, Massimiliano Rossi, Sam Kovaka, Michael C. Schatz, Travis Gagie, Christina Boucher, and Ben Langmead

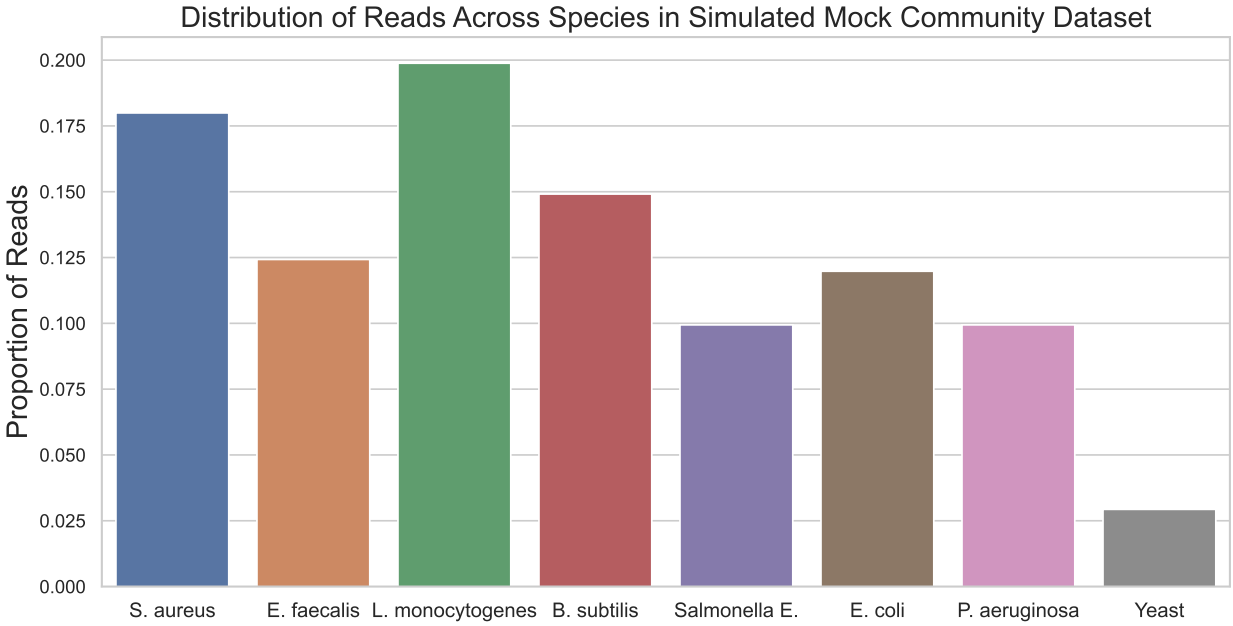

Supplementary Figure 1: Proportion of reads from each species in mock community in the simulated mock community dataset which is related to Section 2.3.2.

**Number of Genomes Included in Each Mock Community Index**		
**Species\Reference:**	One Genome Ref.	Pan-genome Ref.
Staphylococcus aureus	1	574
Enterococcus faecalis	1	49
Listeria monocytogenes	1	225
Bacillus subtitlis	1	165
Salmonella enterica	1	880
Escherichia coli	1	1370
Pseudomonas aeruginosa	1	274
Saccharomyces cerevisiae	1	1

Table S1: Number of genomes for each species in the different references used for the mock community experiment which is related to Section 2.3.2.

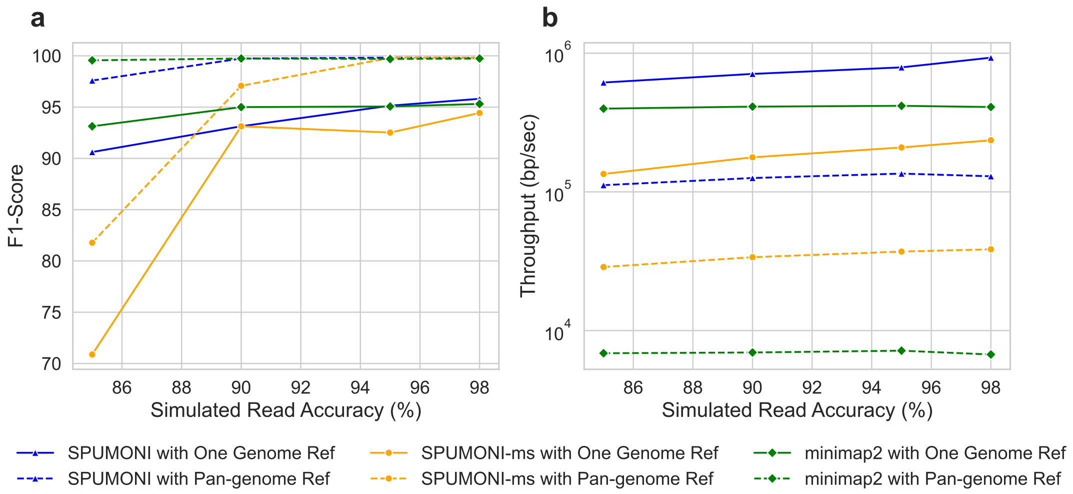

Supplementary Figure 2: Visualization of (a) F_1-score, (b) throughput with varying simulated base-call accuracies which is related to Table 2.

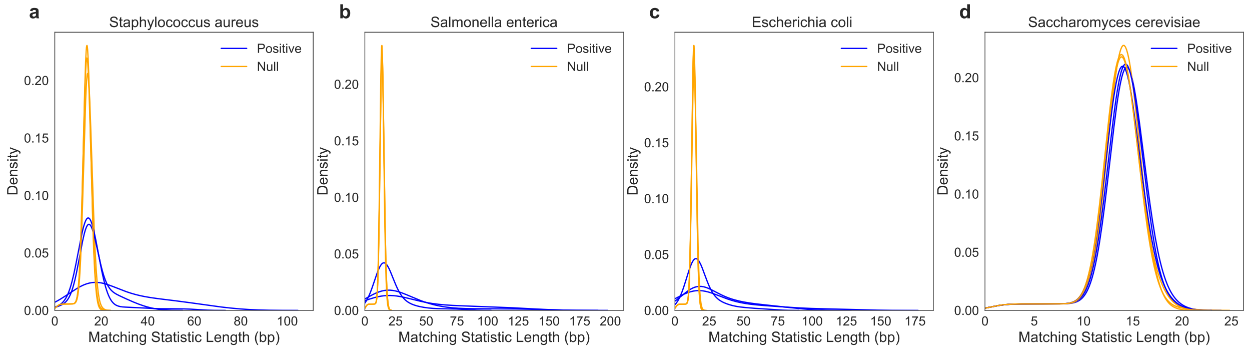

Supplementary Figure 3: Distribution of matching statistics against positive and null indexes for three randomly chosen reads of (a) *Staphylococcus aureus*, (b) *Salmonella enterica*, (c) *Escherichia coli*, and (d) *Saccharomyces cerevisiae*. Each density curve is based on the first 720 bases ($\sim$ 1.6 seconds) of ReadUntil data for each read, which is related to Section 2.3.3.

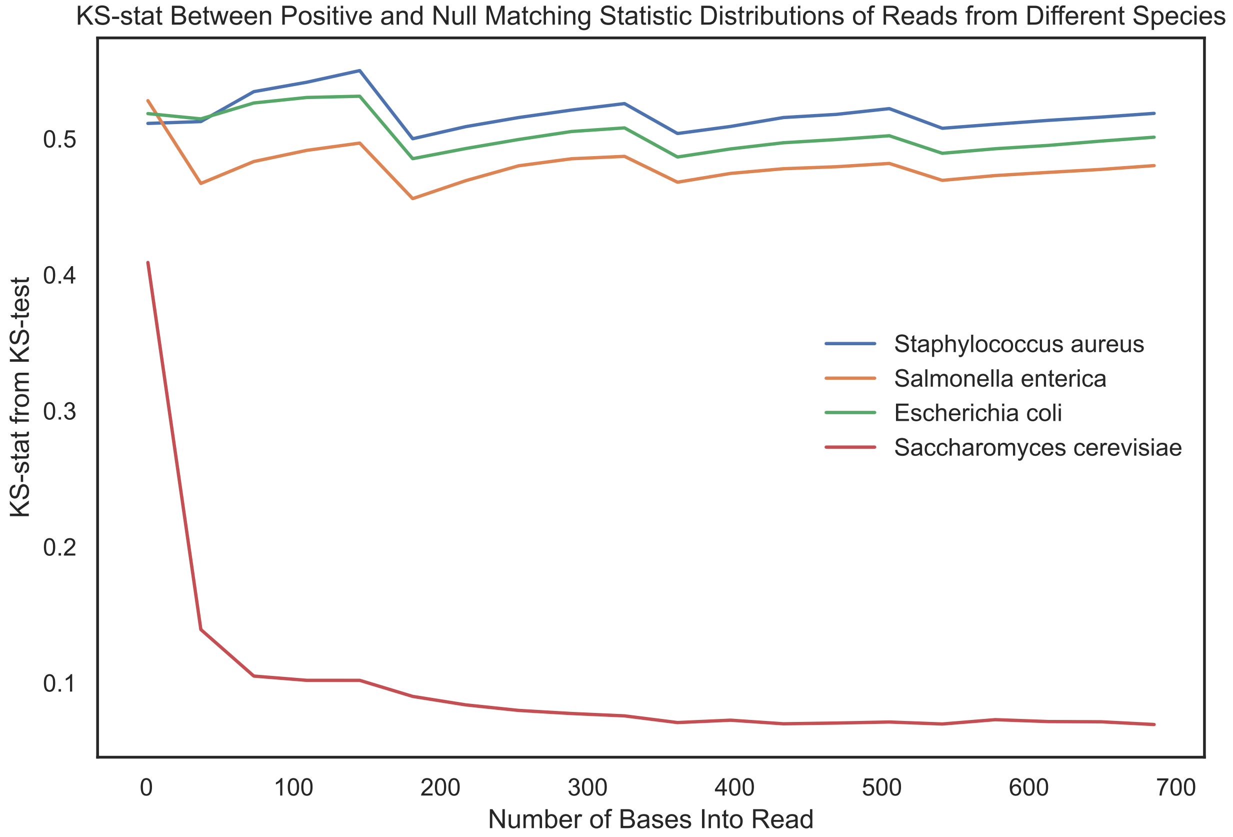

Supplementary Figure 4: Each line represents the average of the Kolmogorov-Smirnov statistic (KS-stat) across all reads from that respective species in the mock community. The figure (related to Section 2.3.3) shows that for bacterial species (*Staphylococcus aureus*, *Salmonella enterica*, and *Escherichia coli*) the KS-stat is relatively larger at ~ 0.5 since the reads are matching to sequence in the positive index. While for the yeast reads, the KS-stat is small at ~ 0.1 since the distribution of positive and null matching statistics are quite similar to each other.

Algorithm 1 Matching Statistics

given text T and a pattern p
//first pass: get pointers
$j \leftarrow 1, pos \leftarrow SA[j]$
for $i \leftarrow p.len$ to 1 **do**
  if $p[i] \neq \mathrm{BWT}[j]$ **then**
    if $j < \mathrm{Thr}(j, p[i])$ **then**
      $j \leftarrow \mathrm{BWT.pred}(j, p[i])$
    else
      $j \leftarrow \mathrm{BWT.succ}(j, p[i])$
    $pos \leftarrow \mathrm{SA}[j] - 1$
  else
    $pos \leftarrow pos - 1$
  $pointers[i] \leftarrow pos$
  $j \leftarrow \mathrm{LF}(j)$
//second pass: get lengths
$\ell \leftarrow 0$
for $i \leftarrow 1$ to $pointers.len$ **do**
  $pos \leftarrow pointers[i] + \ell, j \leftarrow i + \ell$
  while $j < p.len$ **and** $p[j] = T[pos]$ **do**
    $\ell \leftarrow \ell + 1$
    $pos \leftarrow pos + 1, j \leftarrow j + 1$
  $\mathrm{ms}[i] \leftarrow \ell$
  $\ell \leftarrow \max(\ell - 1, 0)$
Return ms

Algorithm 2 Pseudo Matching Lengths

given text T and a pattern p
//first pass: get pointers and pmls
$j \leftarrow 1, \ell \leftarrow 0$
for $i \leftarrow p.len$ to 1 **do**
  if $p[i] \neq \mathrm{BWT}[j]$ **then**
    if $j < \mathrm{Thr}(j, p[i])$ **then**
      $j \leftarrow \mathrm{BWT.pred}(j, p[i])$
    else
      $j \leftarrow \mathrm{BWT.succ}(j, p[i])$
    $\ell \leftarrow 0$
  else
    $\ell \leftarrow \ell + 1$
  $\mathrm{pml}[i] \leftarrow \ell$
  $j \leftarrow \mathrm{LF}(j)$
Return pml

Supplementary Figure 5: Matching statistics (Algorithm 1) and Pseudo Matching Lengths (Algorithm 2) computation using the thresholds (which is related to Section 11.2.2). Given an array a, $a.len$ refers to the length of the array. Given a position j in the BWT, $\mathrm{LF}(j)$ is the LF-mapping, SA is the suffix array sampled at run boundaries, $\mathrm{BWT.pred}(j, c)$ is the position in the BWT of the first character c preceding position j, $\mathrm{BWT.succ}(j, c)$ is the position in the BWT of the first character c following position j, $\mathrm{Thr}(j, c)$ is the position of the threshold for the character c between the run of c preceding and following position j in the BWT. To avoid overloading the notation, we consider the variable pos to be $(pos \mod T.len) + 1$. Furthermore, note that if $\mathrm{BWT.pred}(j, c)$, the threshold value is guaranteed to be smaller than or equals to j, i.e., $\mathrm{Thr}(j, c) = 0$. Similarly, if $\mathrm{BWT.succ}(j, c)$, the threshold value is guaranteed to be greater than j, i.e., $\mathrm{Thr}(j, c) = T.len + 1$.
